# Facial expression recognition is linked to clinical and neurofunctional differences in autism

**DOI:** 10.1186/s13229-022-00520-7

**Published:** 2022-11-10

**Authors:** Hannah Meyer-Lindenberg, Carolin Moessnang, Bethany Oakley, Jumana Ahmad, Luke Mason, Emily J. H. Jones, Hannah L. Hayward, Jennifer Cooke, Daisy Crawley, Rosemary Holt, Julian Tillmann, Tony Charman, Simon Baron-Cohen, Tobias Banaschewski, Christian Beckmann, Heike Tost, Andreas Meyer-Lindenberg, Jan K. Buitelaar, Declan G. Murphy, Michael J. Brammer, Eva Loth

**Affiliations:** 1 Department of Psychiatry and Psychotherapy, University of Frankfurt, Frankfurt, Germany; 2 Department of Forensic and Neurodevelopmental Sciences, Institute of Psychiatry, Psychology and Neuroscience, King’s College London, London, UK; 3 Department of Psychiatry and Psychotherapy, Central Institute of Mental Health, University of Heidelberg/Medical Faculty Mannheim, Mannheim, Germany; 4 Department of Psychology, Social Work and Counselling, Faculty of Education and Health, Greenwich University, London, UK; 5 Centre for Brain and Cognitive Development, Department of Psychological Sciences, University of London, Birkbeck London, UK; 6 Autism Research Centre, Department of Psychiatry, University of Cambridge, Cambridge, UK; 7 Donders Institute for Brain, Cognition and Behavior, Radboud University, Nijmegen, The Netherlands

**Keywords:** Autism, Autism spectrum disorder, Facial expression recognition, Clustering analysis, Stratification biomarkers, Social brain, fMRI, Development, Multi-site

## Abstract

**Background:**

Difficulties in social communication are a defining clinical feature of autism. However, the underlying neurobiological heterogeneity has impeded targeted therapies and requires new approaches to identifying clinically relevant bio-behavioural subgroups. In the largest autism cohort to date, we comprehensively examined difficulties in facial expression recognition, a key process in social communication, as a bio-behavioural stratification biomarker, and validated them against clinical features and neurofunctional responses.

**Methods:**

Between 255 and 488 participants aged 6–30 years with autism, typical development and/or mild intellectual disability completed the Karolinska Directed Emotional Faces task, the Reading the Mind in the Eyes Task and/or the Films Expression Task. We first examined mean-group differences on each test. Then, we used a novel intersection approach that compares two centroid and connectivity-based clustering methods to derive subgroups based on the combined performance across the three tasks. Measures and subgroups were then related to clinical features and neurofunctional differences measured using fMRI during a fearful face-matching task.

**Results:**

We found significant mean-group differences on each expression recognition test. However, cluster analyses showed that these were driven by a low-performing autistic subgroup (~ 30% of autistic individuals who performed below 2SDs of the neurotypical mean on at least one test), while a larger subgroup (~ 70%) performed within 1SD on at least 2 tests. The low-performing subgroup also had on average significantly more social communication difficulties and lower activation in the amygdala and fusiform gyrus than the high-performing subgroup.

**Limitations:**

Findings of autism expression recognition subgroups and their characteristics require independent replication. This is currently not possible, as there is no other existing dataset that includes all relevant measures. However, we demonstrated high internal robustness (91.6%) of findings between two clustering methods with fundamentally different assumptions, which is a critical pre-condition for independent replication.

**Conclusions:**

We identified a subgroup of autistic individuals with expression recognition difficulties and showed that this related to clinical and neurobiological characteristics. If replicated, expression recognition may serve as bio-behavioural stratification biomarker and aid in the development of targeted interventions for a subgroup of autistic individuals.

**Supplementary Information:**

The online version contains supplementary material available at 10.1186/s13229-022-00520-7.

## Introduction

Autism spectrum disorder^1^ (henceforth ‘autism’) is a life-long neurodevelopmental condition, behaviourally defined by difficulties in social communication alongside repetitive and restricted behaviours and interests [1]. Over recent years, the clinical and etiological heterogeneity of autism has been identified as a key barrier for mapping clinical features to underlying mechanisms, and to the development of targeted support or interventions [2]. Several neurocognitive and neurobiological characteristics have been identified at the group level, but none have been found to be either universal (i.e. applying to all autistic people) or specific (i.e. many differences are also seen in other neurodevelopmental or psychiatric conditions [3]) to autism. These observations have prompted recent research efforts to move beyond the analysis of group-level differences to identify tests or markers that can be used to stratify autism into clinically relevant biological subgroups [4]. A stratification biomarker could be any objectively measurable characteristic that can be used to subdivide or stratify a condition into more homogeneous subgroups. It could be specific for a subpopulation within an established clinical category (here autism) or may be transdiagnostic; that is, pertaining to a subpopulation of individuals across diagnostic categories. A stratification biomarker may be constructed from a univariate measure, whereby clinically relevant differences are indicated from certain cut-off values, or derived from multi-variate measures. Availability of validated stratification biomarkers is critical for the development of better targeted interventions.

The current study explored difficulties in facial expression recognition as a potential stratification marker for social communication difficulties in autism. Expression recognition is a plausible candidate marker because the ability to recognise (and respond to) others’ emotional states based on their facial expressions is a fundamental social perceptual skill that modulates many aspects of social interaction and communication. It emerges in infancy [5], becomes more sophisticated across development and has a well-defined neurobiological basis [6].

Difficulties in expression recognition in autism have been reported at both the behavioural and neurobiological level. Overall, findings indicate that the nature and severity of expression recognition difficulties in autism may depend on both participant characteristics (e.g. age, intellectual ability level) and task-related factors [7, 8]. For example, difficulties in recognising *basic* emotions (e.g. happy, sad, fear) have been predominantly found in studies with young autistic children [9, 10] or those with co-occurring learning disabilities. Some behavioural studies reported that adolescents/adults with an IQ in the average range had difficulties on tests assessing complex emotions [11], emotions displayed only briefly [12, 13] or subtle emotions [14]; others found no group differences [15]. Furthermore, neuroimaging studies have revealed neurofunctional differences in autistic individuals during emotion processing tasks in key areas comprising the ‘social brain network’, including the amygdala and right posterior fusiform gyrus (FG) (also known as the ‘fusiform face area’) [16, 17]. The fusiform face area is thought to be involved in the processing of faces [18] and other objects that people have significant expertise with [19], while the amygdala plays a critical role in the processing of emotional and socially relevant information, including the recognition of emotions from facial expressions. The spatial location and direction of effect vary; for example, some studies reported that the autistic group had on average under-activation in the amygdala [20], while others found overactivation in the autistic group [21].

However, to date, the majority of behavioural and neuroimaging studies have focused on mean-group differences, and often included sample sizes of 15–20 individuals per group (see [8]), which are too small to meaningfully subdivide the autism group. Significant difficulties found at the group level, especially with small or moderate effect sizes, may not apply to particular individuals within that group [22]. It is important to ascertain whether only subgroups of autistic individuals have difficulties in expression recognition, and to what extent those difficulties impact clinical features or functional outcomes, in order to better target therapies.

Hence, in this study we explored the role of expression recognition as a candidate stratification biomarker in a large group of autistic individuals diverse in age and intellectual ability. We employed three facial expression recognition tasks: the Karolinska Directed Emotional Faces (KDEF) [23], the Reading the Mind in the Eyes Test (RMET) [24], and the Films Expression task (FET) [13]. First, we carried out case–control comparisons on each task and ascertained the frequency and severity of difficulties. Second, we used clustering approaches to identify subgroups based on combined performance across all three tasks. Here, we performed clustering separately for the autistic and non-autistic groups, with the primary goal of parsing heterogeneity in expression recognition among autistic people. We then repeated the same approach in the control group to ascertain whether similar (or different) patterns may exist in non-autistic individuals.

Finally, to externally validate expression recognition as a potential stratification biomarker, we compared performance on single measures and subgroups in terms of differences in social communication symptoms, functional outcomes, and regional activation measured using fMRI in key areas implicated in emotional face processing [25]. We hypothesised that difficulties in expression recognition would be associated with increased social communication difficulties and neurofunctional differences, and that the combined performance profiles across tasks may be more sensitive than individual task performance. Such a profile could potentially serve as a stratification biomarker in autism.

## Methods and materials

### Participants

Participants were recruited as part of the EU-AIMS Longitudinal European Autism Project (LEAP), a multi-centre longitudinal European initiative seeking to identify biomarkers in autistic people aged 6–30 years [2]. Participants completed a battery of clinical, cognitive, EEG and MRI assessments at one of the following six centres: Institute of Psychiatry, Psychology and Neuroscience, King’s College London, UK; Autism Research Centre, University of Cambridge, UK; Radboud University Nijmegen Medical Centre, the Netherlands; University Medical Centre Utrecht, the Netherlands; Central Institute of Mental Health, Mannheim, Germany; University Campus Bio-Medico of Rome, Italy. The study was approved by the respective research ethics committees at each site (IRAS, UK). Informed written consent was obtained from all participants, or—for minors or those unable to give informed consent—from a parent or legal guardian.

Participants were sorted into cohorts based on age and intellectual ability: adults between 18–30 years (schedule A), adolescents between 12–17 years (schedule B), children between 6–11 years (schedule C)—all with IQ in the normal range—and adolescents/adults with mild intellectual disability (ID) (IQ 50–74) between 12–30 years (schedule D). For further details about the study design and clinical characterisation of the cohort, see Loth et al. [2] and Charman et al. [26]. The current study reports on a subset of measures acquired at the follow-up assessment time-point (2016–2018), as two facial expression tests were only added to the protocol at this time.

In the following analyses, we compared the autism group (including autistic individuals with mild ID, defined by FSIQ < 75) to a ‘control group’, comprising individuals with typical development (TD) and mild intellectual disability (ID, defined by IQ < 75). We also divided each group by study schedule as outlined above to investigate whether group differences may be greater at certain developmental stages (e.g. in children vs. adults), or in individuals with mild ID.

Participants in the autism group had an existing clinical diagnosis of autism spectrum disorder according to the DSM-IV-TR/ICD-10 or DSM-5 criteria at study entry. For research purposes, we also conducted a structured parent interview—the Autism Diagnostic Interview-Revised (ADI-R; [27]), which combines historic and current symptoms; and administered the Autism Diagnostic Observation Schedule 2 (ADOS-2; [28]) to participants, which measures current behaviours. We used the ADOS Calibrated Severity Score (CSS) for Social Affect (SA) as a measure of current social symptoms.

To assess autism traits, the parent-reported total t-score on the Social Responsiveness Scale Second Edition (SRS-2; [29]) was used in all participants except for TD adults, where the self-report version was used. The Repetitive Behavior Scale-Revised (RBS-R; [30]) was used to assess the severity of repetitive behaviours and the Vineland Adaptive Behavior Scales-2nd Edition (VABS; [31] social adaptive behaviour subscale) to measure social adaptive functioning. Neuropsychiatric symptoms were measured using the Development and Wellbeing Assessment (DAWBA) probability bands [32].

We examined the most common neuropsychiatric symptoms in autism (anxiety, depression, ADHD, behavioural disorder) and generated a total ‘neuropsychiatric symptom score’ based on all disorders. DAWBA scores reflect six levels of prediction (i.e. ~ 0.1%, ~ 0.5%, ~ 3%, ~ 15%, ~ 50%, > 70%) of the probability of a disorder, ranging from very unlikely to probable. We derived a binary measure of predicted DAWBA risk by combining Levels 0–3 as ‘absent’ (i.e. having a risk score of ~ 0.5%, ~ 3%, ~ 15%) and Levels 4–5 as ‘present’ (~ 50%, > 70%). In the autism and ID groups, all neuropsychiatric symptoms were allowed. An inclusion criterion for participants in the ‘typically developing’ control group was the absence of any existing clinical diagnosis (including mental health problems). This contributed to the relatively low rate of psychiatric/mental health features in the control group. Medication use was assessed using an in-house questionnaire.

### Facial expression recognition tasks

The *Karolinska Directed Emotional Faces task (KDEF)* [23] tests for recognition of six basic emotions (happy, sad, angry, fearful, surprised, disgusted) and uses long presentation times (20 s).

The *Reading the Mind in the Eyes Task (RMET)* [24] requires participants to identify complex emotions based only on the eye region of a face. Depending on age, participants received either an adult (36 items), adolescent (31 items), or child (28 items) version of the test.

**Fig. 1:**
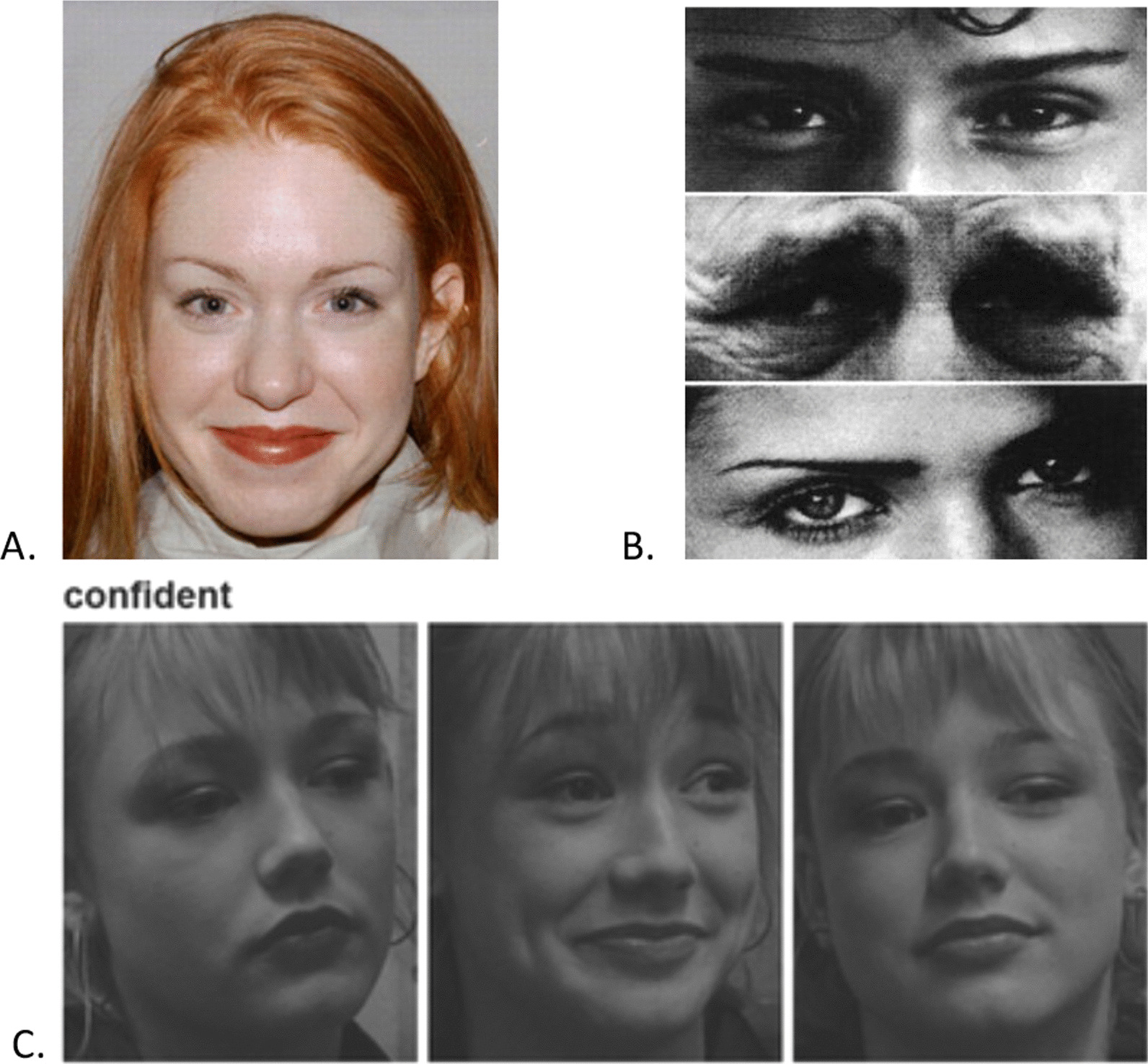
Example stimuli from all three facial recognition tasks. **A** KDEF, **B** RMET, and **C** FET

The *Films Expression Task (FET)* [13] tests for recognition of both basic and complex emotions from film stills and has short presentation times (500 ms). Depending on age and ability level, participants received an adult version with 56 items (or a child version, created for LEAP) with a subset of 36 items that only employed emotion terms that children within the targeted age range typically understand. Examples of test stimuli for all three tasks are given in Fig. 1. More details of each task characteristics, procedures, control tests and reliability information are provided in Additional file 1: Materials 1.

Across all three tasks, accuracy and accuracy-adjusted reaction times (ART, the mean time of each passed trial divided by the fraction of passed trials) were used as the main dependent variables.

We examined group differences overall as well as in each age group (corresponding to different task versions) in order to see whether difficulties were more pronounced at some developmental stages than in others.

### Group comparisons

To investigate case–control differences in response accuracy, we used nonparametric Kruskal–Wallis tests, as data distributions were skewed towards high performance across tests (Shapiro–Wilk normality tests: KDEF: *p* < 0.001; RMET: *p* < 0.001; FET: *p* < 0.001), and nonparametric ANOVA (fANCOVA), controlling for the effects of VIQ and neuropsychiatric symptoms (see Results) on task performance. To investigate case–control differences in ART, ANOVAs or ANCOVAs were used for all three tasks. Age was not controlled for as two of the tasks used different versions for different age groups, so we report findings split by age groups. We also did not control for sex but tested for the effect of sex on each task.

We used Bonferroni corrections to correct for the number of tests (i.e. 0.05/6 = 0.008).

Spearman’s correlations (and partial correlations, controlling for the previously documented effect of VIQ on expression recognition) were used to examine dimensional relationships of each expression recognition task to clinical features or ROIs (regions of interest in neuroimaging, see below). All analyses were performed in Rstudio Version 1.1.453 2009–2018. The number of participants varied between tasks. All details are provided in Table 1 (See below).

**Table 1:** Participant characteristics, by task

	*N*		Age (yrs) Mean ± SD Range			IQ Mean ± SD Range			Sex m:f			Neuropsychiatric symptoms*		Medication use	
	Autism	Control^1^	Autism	Control	Sign	Autism	Control	Sign	Autism	Control	Sign	Autism	Control	Autism	Control
	*A. KDEF*														
Overall	251	150	16.38 ± 5.78 6.23–30.17	15.68 ± 5.68 6.23–29.8	*P* > 0.05	98.69 ± 19.88 55–148	104.49 ± 19.23 51–142	***P* < 0.01**	181:70	98:52	*P* > 0.05	99	4	108 (43%)	29 (19%)
Schedule A	70	41	22.48 ± 3.53 18.01–30.13	22.31 ± 3.28 18.11–29.84	*P* > 0.05	105.39 ± 14.95 76–148	111.49 ± 13.06 76–142	***P* < 0.05**	52:18	29:12	*P* > 0.05	20	0	28 (40%)	8 (20%)
Schedule B	80	42	15 ± 1.82 12.06–17.89	15.1 ± 1.57 12.24–17.67	*P* > 0.05	102 ± 16.66 75–143	105.98 ± 12.92 77–126	*P* > 0.05	62:18	28:14	*P* > 0.05	33	1	37 (46%)	7 (17%)
Schedule C	61	48	9.37 ± 1.49 6.23–11.96	9.39 ± 1.6 6.23–11.97	*P* > 0.05	107.02 ± 14.44 77–148	112.6 ± 11.69 85–142	***P* < 0.05**	41:20	28:20	*P* > 0.05	31	0	26 (43%)	5 (10%)
Schedule D	40	19	19.15 ± 5.1 11.49–30.17	18.53 ± 3.91 12.58–27.11	*P* > 0.05	67.03 ± 5.34 55–74	65.58 ± 7.45 51–74	*P* > 0.05	26:14	13:6	*P* > 0.05	15	3	17 (43%)	9 (47%)
	*B. RMET*														
Overall	277	211	16.45 ± 5.75 6.23–30.26	16.35 ± 5.65 6.23–30.95	*P* > 0.05	101.9 ± 18.43 56–148	107.21 ± 16.28 52–142	***P* < 0.001**	198:78	134:77	*P* > 0.05	104	8	120 (43%)	38 (18%)
Schedule A	87	67	22.57 ± 3.51 18.01–30.26	22.51 ± 3.42 18.11–30.95	*P* > 0.05	105.87 ± 15.01 76–148	110.88 ± 12.93 76–142	***P* < 0.05**	61:26	42:25	*P* > 0.05	24	1	34 (39%)	14 (21%)
Schedule B	89	74	15.05 ± 1.83 12.06–17.89	15.38 ± 1.72 12.03–17.96	*P* > 0.05	105.02 ± 14.8 78–143	107.05 ± 12.61 77–140	*P* > 0.05	69:20	50:24	*P* > 0.05	35	4	41 (46%)	12 (16%)
Schedule C	70	57	9.44 ± 1.54 6.23–11.96	9.67 ± 1.58 6.23–11.97	*P* > 0.05	107.61 ± 14.87 77–148	112.49 ± 11.7 85–142	***P* < 0.05**	49:21	34:23	*P* > 0.05	32	0	32 (46%)	5 (9%)
Schedule D	31	13	19.08 ± 4.24 12.77–29.21	19.8 ± 3.9 14.55–27.11	*P* > 0.05	66 ± 7.45 52–74	68.13 ± 4.63 56–74	*P* > 0.05	19:11	8:5	*P* > 0.05	13	3	13 (42%)	7 (54%)
	*C. FET*														
Overall	148	107	16.31 ± 5.75 6.23–28.57	15.88 ± 5.99 7.43–30.21	*P* > 0.05	100.08 ± 19.27 55–148	105.52 ± 19.28 52–142	***P* < 0.05**	102:46	72:35	*P* > 0.05	56	5	70 (47%)	22 (21%)
Schedule A	47	30	22.4 ± 3.47 18.02–29.57	22.72 ± 3.28 18.11–29.84	*P* > 0.05	105.22 ± 16.04 76–148	112.6 ± 11.15 94–142	***P* < 0.05**	32:15	22:8	*P* > 0.05	13	0	22 (47%)	5 (17%)
Schedule B	48	28	14.99 ± 1.88 12.28–17.89	15.03 ± 1.70 12.24–17.67	*P* > 0.05	101.38 ± 16.18 75–142	107.68 ± 11.79 77–126	*P* > 0.05	36:12	19:9	*P* > 0.05	21	1	26 (54%)	4 (14%)
Schedule C	35	36	9.16 ± 1.62 6.23–11.91	9.5 ± 1.39 7.43–11.69	*P* > 0.05	107.6 ± 14.94 77–148	113 ± 12.24 85–142	*P* > 0.05	22:13	23:13	*P* > 0.05	14	0	14 (40%)	5 (14%)
Schedule D	18	13	17.89 ± 4.82 13.79–27.21	19.56 ± 4.79 13.79–30.21	*P* > 0.05	67 ± 5.47 55–74	63.85 ± 7.01 52–74	*P* > 0.05	12:6	8:5	*P* > 0.05	8	4	8 (44%)	8 (61%)

Statistical significance was defined as p-value of 0.05 divided by the number of tests performed

^1^For Schedules A-C (defined by age and IQ: A - > children, B - > adolescents, C - > adults, IQ ≥ 75 the control group comprises typically developing participants, for Schedule D (defined by age and IQ) those are adults and adults from 12 years with mild ID (IQ 50–75)

*Neuropsychiatric symptoms that data was gathered on included ADHD, anxiety, depression, and behavioural disorders

### Clustering analysis

Clustering analyses were carried out in 129 autistic and 96 control participants who had completed all three behavioural expression recognition tasks. Cluster analysis was carried out using both hierarchical clustering (Ward’s linkage, Euclidean distance) and K-means (25 random starts). These two methods were chosen because they represent the most common variants of centroid and connectivity-based clustering, respectively, which are based on different assumptions and tend to produce the most divergent results. Here we carried out clustering separately for the autism and control groups, as our main goal was to examine heterogeneity among autistic individuals. More details on the clustering analysis are given in Additional file 1: Materials 2.

### fMRI procedures

We had valid data of a subset of 132 participants from a well-established emotional face-matching task, the Hariri task, while fMRI blood-oxygen-level-dependent (BOLD) responses were recorded [2]. As per LEAP study protocol [2], this task was mainly administered to schedules A and B to reduce scan time for children and those with mild ID. There were no significant differences between the sub-sample with fMRI data and the overall sample in terms of age or sex composition, but the sub-sample with fMRI data had on average significantly higher IQ (*p* < 0.001).

Please see Additional file 1: Materials 3 for more information on the fMRI paradigm, data acquisition, and analysis.

## Results

### Case–control comparisons

Participant characteristics for each task are given in Table 1.

#### Preliminary analyses

First, we examined the effect of study site, schedule (and separately age and IQ), sex, medication, and neuropsychiatric symptoms on expression recognition, as detailed in Table 1. There were no significant effects of study site or medication on any accuracy scores or ART (all *p* > 0.05). However, there were significant effects of schedule on all tests (all *p* < 0.001). In both the autism and control groups, performance on each expression recognition task was moderately correlated with verbal, performance, and full-scale IQ (autism *r*’s: 0.34–0.53, control group *r*’s: 0.24–0.44, all *p* < 0.001). Age was weakly correlated with accuracy on the KDEF (*r* = 0.15, *p* < 0.05) in the autism group, but not significantly in the control group (*r* = 0.11, *p* > 0.05); presumably because the task employed expressions of basic emotions and long presentation times, and age effects may only occur in younger children than those tested in this cohort. There were no age effects on the RMET and FET, which employed different age-related versions.

Sex had no significant effect on the KDEF (*p* > 0.05), but we found nominally significant sex differences on the FET (*p* < 0.05) and a non-significant trend on the RMET (*p* = 0.06), such that males performed marginally worse than females. In the autism group, there was no significant difference between those with and without neuropsychiatric symptoms on any task performance (all *p* > 0.05). In the control group, we found a significant effect of neuropsychiatric symptoms on the RMET (*p* < 0.05, *r* =  − 0.18) and the FET (*p* < 0.01, *r* = 0.31). This effect was mainly driven by a small number of participants with ID, as any psychiatric disorder was an exclusion criterion in the ‘typically developing’ group (8 in the RMET and 5 in the FET); hence, the following analyses were performed overall, and split by schedule to compare more homogeneous age/IQ groups, and with/without controlling for IQ and neuropsychiatric symptoms. The results of preliminary analyses are summarised in Table 2.

**Table 2:** Correlations between performance on each expression recognition task and participant characteristics, symptom severity, and level of adaptive behaviour, by group (*N* in brackets)

	Autism			Control		
	KDEF	RMET	FET	KDEF	RMET	FET
VIQ	0.36**** (249)	0.48***** (270)	0.50**** (146)	0.24*** (150)	0.21** (210)	0.44**** (107)
PIQ	0.34**** (250)	0.42**** (272)	0.47***** (147)	0.0.38**** (150)	0.25*** (210)	0.27** (107)
FSIQ	0.37**** (249)	0.50***** (270)	0.53**** (146)	0.32*** (150)	0.24*** (210)	0.39*** (107)
Age	0.15* (261)	− 0.03 (276)	0.11 (148)	0.11 (150)	− 0.03 (210)	− 00.1 (107)
SRS-2,	− 0.19** (214)	− 0.22** (239)	− 0.33**** (126)	− 0.20 (87)	− 0.15 (117)	− 0.49*** (63)
ADOS, CSS	− 0.30**** (186)	− 0.39***** (204)	− 0.51***** (105)	–	–	–
RBS-R	− 0.19 (212)	− 0.10 (232)	− 0.28*** (123)	− 0.25 (88)	− 0.12 (114)	− 40** (64)
VABS-2	0.21** (215)	0.22** (230)	0.36**** (126)	0.64** (16)	0.56** (15)	0.57* (13)
Neuropsychiatric symptoms	− 0.12 (251)	0.02 (276)	− 0.08 (148)	− 0.13 (150)	− 0.18* (211)	− 0.31** (107)
Medication use	0.11 (238)	− 0.01 (264)	0.05 (146)	− 0.15 (132)	− 0.04 (198)	− 0.15 (99)

NB. Note that VABS—social adaptive behaviour subscale, was only available for individuals with ID (not TD); Correlations with RBS-R score should be interpreted with caution in the control group, due to skewed scores. ADOS was only available in the autism group,

*p < 0.05, **p < 0.01, ***p < 0.001, ****p < 0.0001, *****p < 0.00001

*KDEF.* The autism group had, on average, significantly lower accuracy scores than the comparison group (*χ*^2^ = 15.17, *p*_corrected_ < 0.001, *r* = 0.18). Analyses split by schedule showed significant differences in autistic adults (*χ*^2^ = 8.8, *p*_corrected_ < 0.05, *r* = 0.26) and children: (*χ*^2^ = 16.01, *p*_corrected_ < 0.01, *r* = 0.34); but not in adolescents: (*χ*^2^ = 3.2, *p* > 0.05) or those with ID: (*χ*^2^ = 0.4 *p* > 0.05). Furthermore, autistic participants were on average significantly less accurate than the comparison group in identifying expressions denoting anger (*χ*^2^ = 21.02, *p*_corrected_ < 0.001, *r* = 0.25), disgust (*χ*^2^ = 13.44, *p*_corrected_ < 0.01, *r* = 0.18) and surprise (*χ*^2^ = 8.38, *p*_corrected_ < 0.05, *r* = 0.16), but not fear. ART did not differ between the two groups overall, or for any specific type of emotion. Overall, between-group differences remained significant after controlling for neuropsychiatric symptoms (all *p* < 0.001).

*RMET***.** On the RMET, we observed significantly reduced average response accuracy in the autism group overall (*χ*^2^ = 22.25, *p*_corrected_ < 0.01, *r* =  − 0.23). When split by schedule the mean-group effect for adults and children was only significant at the uncorrected level (adults: *χ*^2^ = 5.68, *p* < 0.05, *r* = 0.24; adolescents: *χ*^2^ = 6.47, *p* < 0.05, *r* = 0.2; children: *χ*^2^ = 0.64, *p* < 0.05, *r* = 0.21) and non-significant for adolescents/adults with mild ID (*χ*^2^ = 1.48, *p* < 0.05). There were no significant group differences in ART overall, or for any subgroup (all *p* > 0.05), except between those with mild ID (*χ*^2^ = 5.29, *p*_corrected_ < 0.05, *r* = 0.4) with autistic participants showing faster ART than non-autistic participants with mild ID. Overall between-group differences remained significant after controlling for VIQ and neuropsychiatric symptoms (all *p*_corrected_ < 0.05).

*FET.* Overall, there was a significant mean-group difference in accuracy on the FET (*χ*^2^ = 11.59, *p*_corrected_ < 0.001, *r* = 0.24). However, this difference was driven by the adults (*χ*^2^ = 11, *p* < 0.01, *r* = 0.43), with no other schedules showing significance, and became non-significant after controlling for VIQ and neuropsychiatric symptoms (*p* > 0.05). Autistic adults also had on average slower reaction time on accurate trials than non-autistic adults (*χ*^2^ = 9.18, *p*_corrected_ < 0.05, *r* = 0.36).

When analyses were split between trials with simple versus complex emotions, we found significant group differences between autistic and control adolescents and adults in basic emotions (adults: *χ*^2^ = 10.59, *p*_corrected_ < 0.05, *r* =  − 0.36; adolescents: *χ*^2^ = 5.8, *p*_corrected_ < 0.05, *r* = 0.30) and only nominally significantly in the adults in complex emotions (*χ*^2^ = 9.44, *p* < 0.05, *r* =  − 0.23). Autistic children did not differ from comparison children in recognition accuracy for basic or complex emotions, nor did participants with mild ID (all *p* > 0.05).

On the visual processing control task there were no significant group differences in response accuracy (*χ*^2^ = 0.064, *p* > 0.05) or ART (*χ*^2^ = 0.90, *p* > 0.05), which rules out the possibility that performance differences on the FET were due to more general difficulties in processing briefly presented stimuli.

**Fig. 2:**
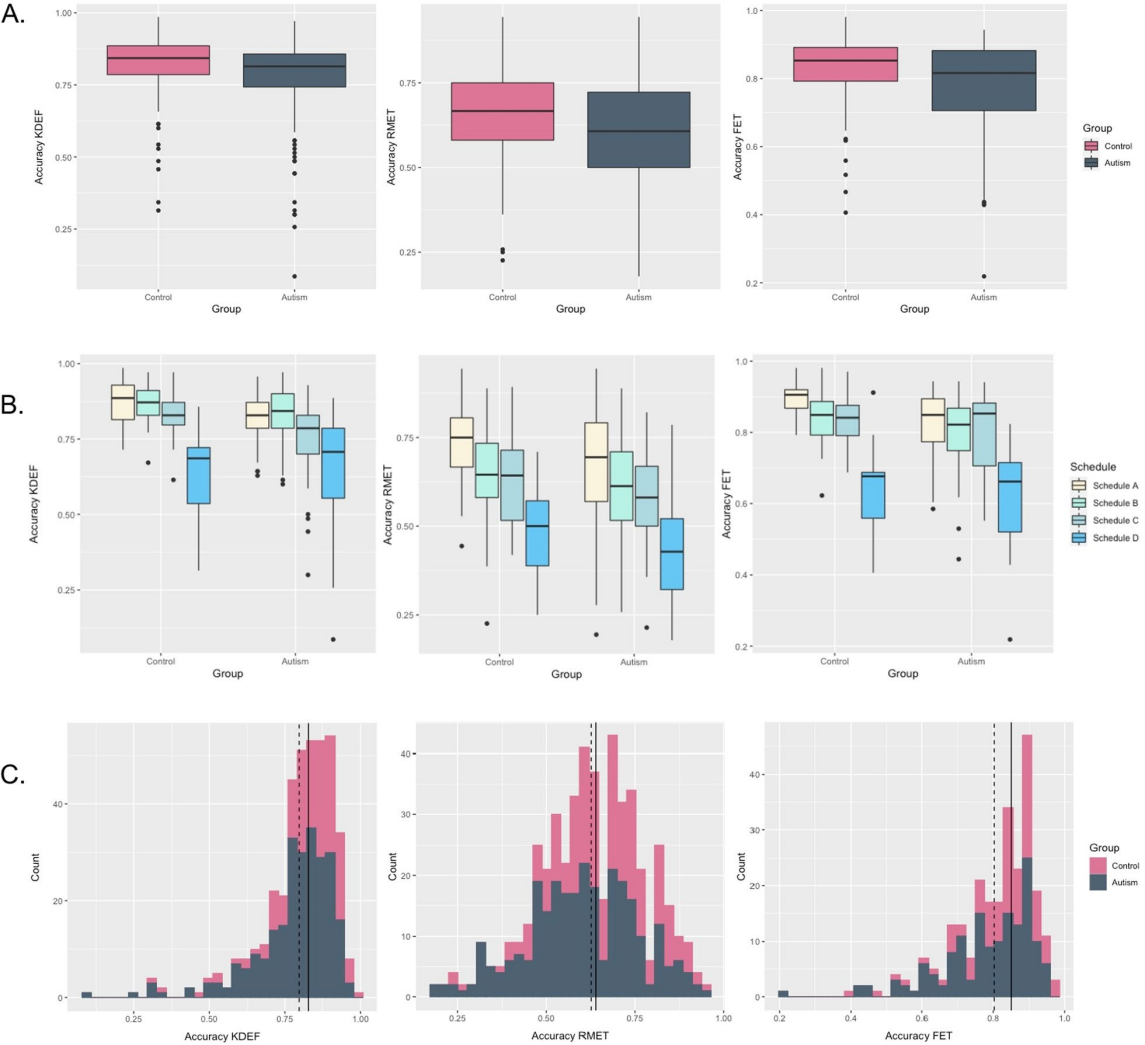
**A** Boxplots showing % accuracy on each task, by diagnostic group [autism, control group. The control group comprises individuals with typical development (TD) and intellectual disability]. **B** Boxplots showing % accuracy on each task, by diagnostic group and age group. Beige = Schedule A, adults, turquoise = Schedule B, adolescents, light blue = Schedule C, children, and dark blue = Schedule D, individuals with ID. **C**. Histograms, showing distributions per group. Solid line indicates 1SD below the control group’s overall median; dotted line indicates 2SDs below the median

Case–control findings are summarised in Fig. 2.

Additional file 1: Materials 4 shows that in both groups, the accuracy measures (and ART measures) were moderately positively correlated with one another. Accuracy and ART were only negatively correlated on the FET, indicating that on this task difficulties in correctly identifying expressions were accompanied with longer response times and vice-versa.

### Clustering analysis

Table 3 gives participant details of the subset of participants who had completed all three expression recognition tasks and were included in the clustering analyses.

**Table 3:** Participant details of participants included in the clustering analyses

	Autism		Control	
	Cluster 1 *N* = 83	Cluster 2 *N* = 36	Cluster 3 *N* = 66	Cluster 4 *N* = 24
Age	16.62 ± 5.69 7.47–29.57	15.45 ± 6.52 6.23–28.74	16.72 ± 6.23 7.55–29.84	14.44 ± 4.82 7.68–27.11
VIQ	109.22 ± 16.13 66–160	85.6 ± 17.38 45–121	113.81 ± 13.98 89–160	96.25 ± 25.66 51–138
PIQ	109.27 ± 15.15 75–148	88 ± 20.27 53–132	111.77 ± 13.25 85–136	93.67 ± 25.9 49–139
ADOS	4.69 ± 2.43 1–9	6.84 ± 2.54 2–10	N/A	N/A
SRS	69.33 ± 12.7 44–90	77.33 ± 10.46 53–90	44.31 ± 5.69 37–66	51.68 ± 12.75 38–76
RBS	14.22 ± 12.87 0–54	24.03 ± 15.46 1–60	1.06 ± 2.49 0–12	3.42 ± 5.36 0–17
VABS-2	78.75 ± 15.43 20–110	66.82 ± 20.25 20–115	123.5 ± 9.69 117–141	51.25 ± 31.6 28–96
Sex m:f	51:32	29:7	40:26	20:4
ID participants	1 (1.2%)	10 (27.7%)	0 (0%)	8 (33.3%)
Neuropsychiatric symptoms*	29 (34.9%)	17 (47.2%)	0 (0%)	3 (12.5%)
ID + Neuropsychiatric symptoms*	0 (0%)	5 (13.9%)	0 (0%)	3 (12.5%)
Medication use	39	18	11	6

We repeated between-group comparisons on each expression recognition test in this group and found similar patterns to the overall group in case–control comparisons. The autism group had significantly lower accuracy in the RMET (*r* =  − 0.22, *p* < 0.01) and the FET (*r* =  − 0.28, *p* < 0.001), though not in the KDEF (*p* > 0.05), our test for basic emotions.

Cluster analyses were carried out separately for the autism and control groups using the accuracy scores of each test. For each group, a two-cluster solution was found. Further, 91.6% of participants were found in the same clusters by hierarchical clustering and *K*-means, while 8.4% were inconsistently clustered.

**Fig. 3:**
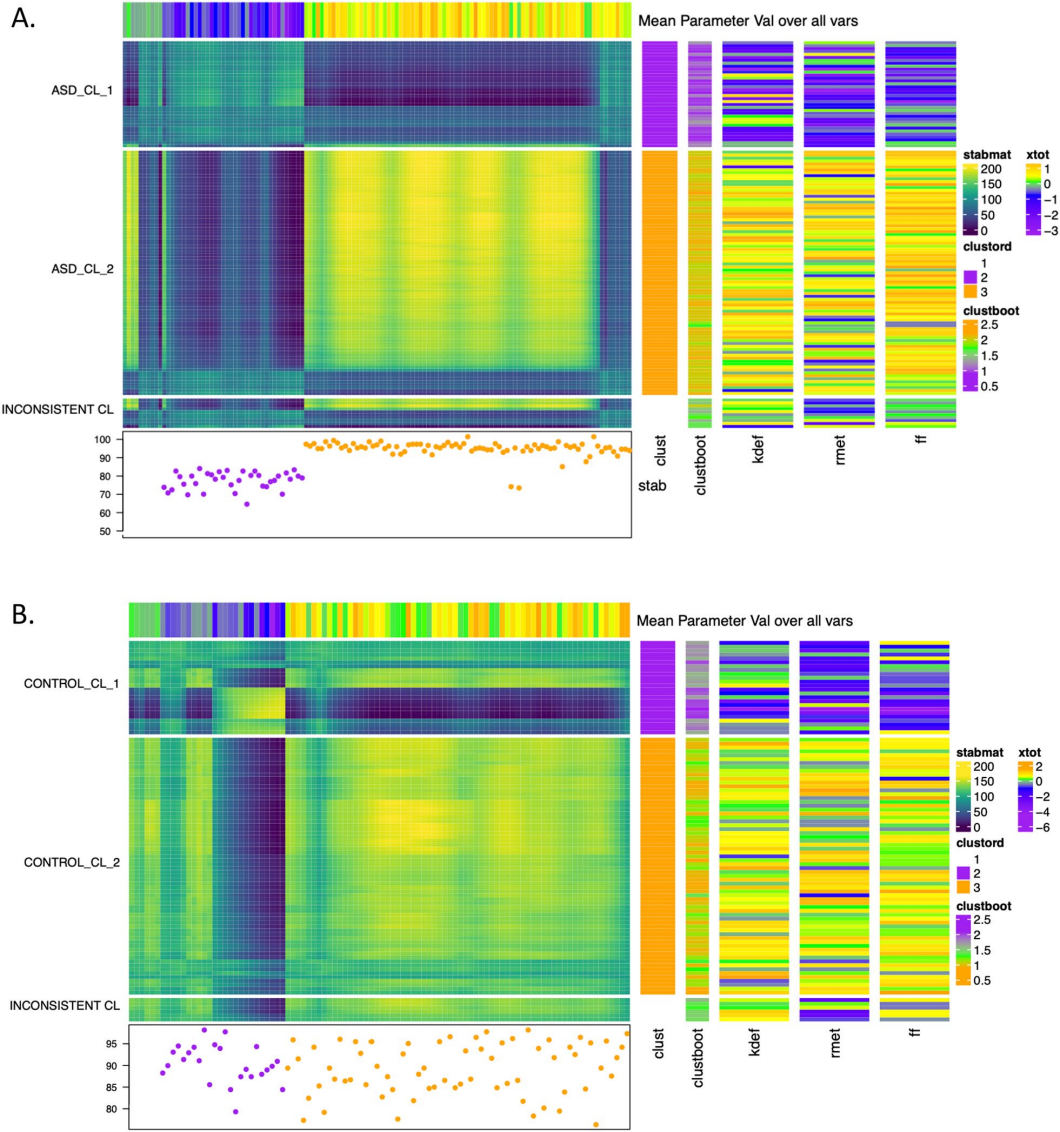
Cluster heat map plus additional annotations, produced by the Superheat package (R), for the autism (**A**) and control groups (**B**)

In the autism group, the larger cluster comprised *n* = 83 (69.7%) individuals and was subsequently characterised by ‘high expression recognition’ performance; the smaller cluster comprised 36 (30.3%) individuals and was characterised by ‘low expression recognition’ performance (see Fig. 3).

Ten of the 36 autistic individuals (27.7%) in the low-performing cluster also had ID, and 17 (47.2%) had neuropsychiatric symptoms. Twenty-nine (75%) were male 12 were children (33.3%), 7 adolescents (19.4%) and 17 adults (47.2%).

The high-performing autistic cluster included only 1 participant with ID but 29 (34.9%) with neuropsychiatric symptoms. Fifty-one (61.1%) were male; 21 were children (25.3%), 29 adolescents (34.9%) and 33 adults (39.8).

This suggests that in the autism group, expression recognition difficulties persisted across all IQ levels: although almost all autistic individuals with ID fell in the low-performing cluster, the majority of autistic low performers actually had IQ in the normal range. There was no significant difference between autistic high- and low-expression recognition performers in terms of neuropsychiatric symptoms.

In the control group, 66 (73.3%) fell in the high expression recognition cluster and 24 (26.7%) in the low expression recognition cluster. In the low-performing cluster, 8 individuals (33.3%) also had ID; 3 (12.5%) had neuropsychiatric symptoms. Both autistic and non-autistic individuals in the low-performing clusters had on average significantly lower verbal, performance, and full-scale IQ than individuals in the high-performing clusters (all *p* < 0.001).

To examine individual task performance within each cluster, we used age-adjusted medians of the typically developing group (excluding those with ID) as a reference point. In the autism high-performing expression recognition cluster, the majority of participants performed above or within one SD of the age-group-related median (KDEF: 82%, RMET: 78.3%, FET: 82%). In the autism low-performing cluster, the majority of participants performed below 2 SD of their age-group-related median (KDEF: 58.3%, RMET: 58.3%, FET: 66.7%). In the control high-performing expression recognition cluster, the majority of individuals performed at or above their age-group-related median in all three tasks (KDEF: 63.6%, RMET: 74.2%, FET: 53%). However, only approximately a third of participants in the low-performing control cluster performed below 2 SD of their age-group-related median (KDEF: 37.5%, RMET: 29.2%, FET: 29.2%). This indicates that the cluster boundaries were more stringent in the autism than control group.

Interestingly, individuals in the ‘non-consistent’ clusters were predominantly characterised by low performance on the RMET but good performance on the KDEF and FET, which may indicate a potential separate subgroup (see Fig. 3A, B, bottom).

### Associations between expression recognition measures/clusters and clinical features

#### Dimensional relationships

**Fig. 4:**
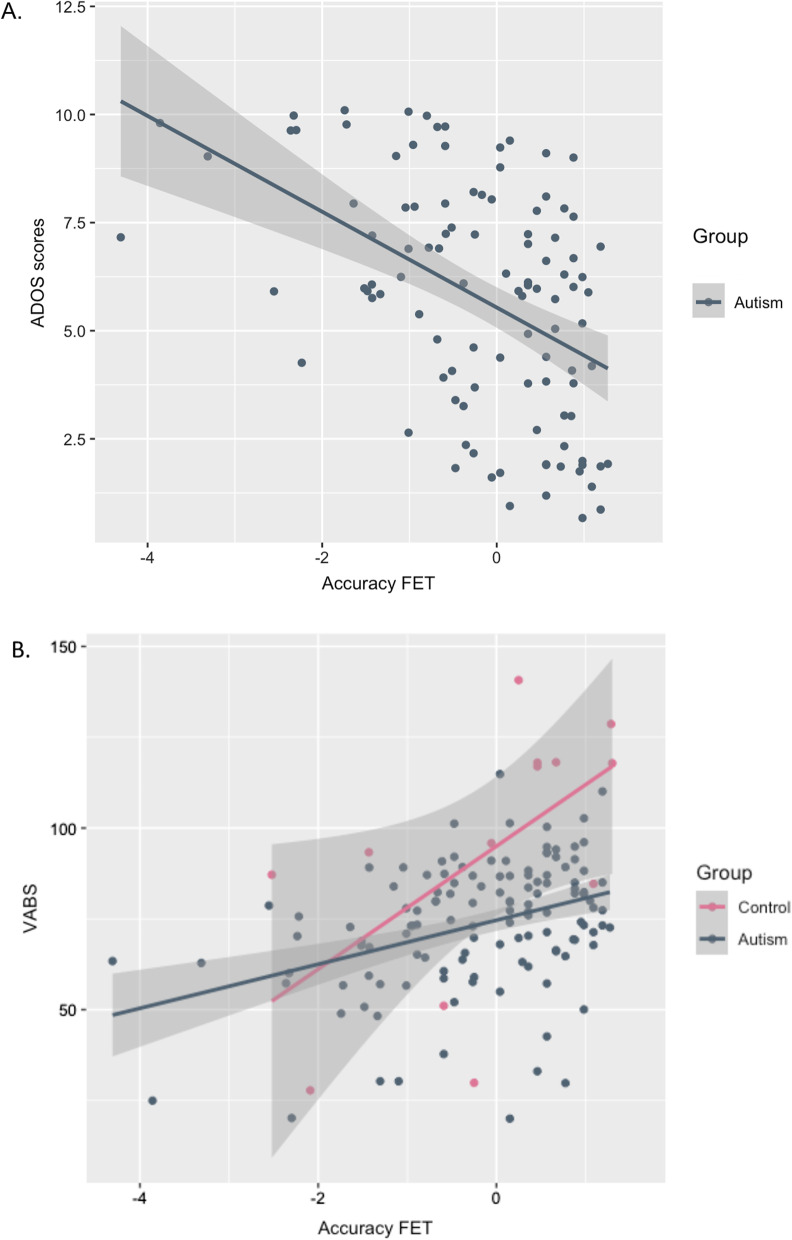
Relationship between FET accuracy and clinical characteristics **A** Scatterplot showing association between accuracy on the FET and current autism symptoms (ADOS scores). **B** Scatterplot showing association with VABS-2 social adaptive behaviour. ADOS, CSS scores were only available for the ASD group; VABS-2 only for the autism group, and among the control group children with TD (Schedule C) or individuals with ID (Schedule D)

Table 3 shows participant characteristics of the individuals included in clustering analysis, including correlations of accuracy on each task to clinical features and social adaptive functions. In the autism group, performance on each expression recognition test was significantly related to current social symptom severity (ADOS, CSS) as well as level of social adaptive functioning (VABS-2; all *r*’s: 0.19–0.36, *p* < 0.01). Repetitive behaviours (RBS-R) only significantly correlated with the FET (*r* =  − 0.28, *p* < 0.01). The strongest associations were found with the FET throughout, which remained significant after the effect of VIQ was controlled for (all *r*’s: − 0.29 to 0.21, *p* < 0.05). In the control group, only FET performance was significantly related to autism traits, which again remained stable after controlling for VIQ (see Fig. 4A, B).

#### Clusters

**Fig. 5:**
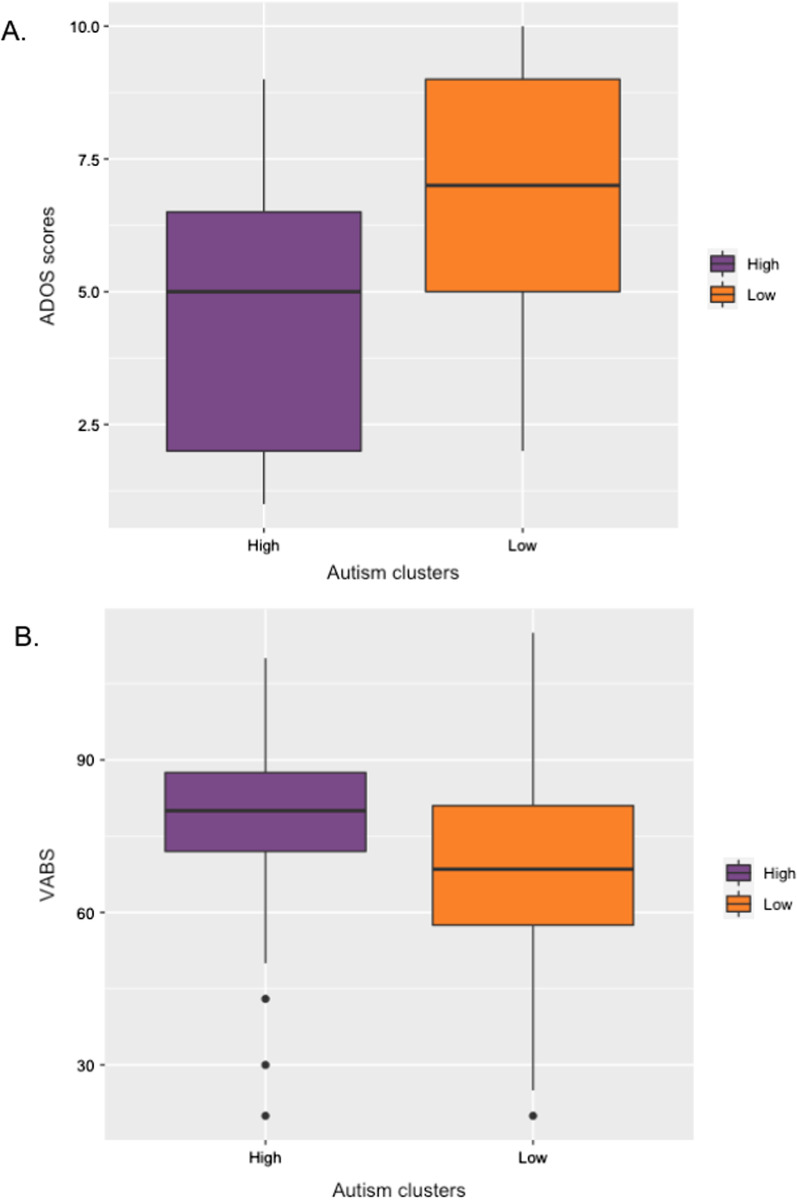
Relationship between clusters and clinical characteristics. **A** Autism cluster differences in autism symptoms (ADOS scores). **B** Autism cluster differences in VABS-2 social adaptive behaviour

When the high- and low-performing autism clusters were compared to each other in terms of clinical features, we found significant subgroup differences in severity of current social symptoms (*t*(88) =  − 3.9, *p* < 0.001, *r* = 0.39), autism traits (*t*(100) = 3.39, *p* < 0.001, *r* = 0.31), repetitive behaviours (*t*(96) = 3.28, *p* < 0.001, *r* = 0.33), and level of social adaptive functioning (*t*(103) = 3.39, *p* < 0.001, *r* = 0.33) (see Fig. 5). Subgroups explained 14.8% of variance in current social symptoms, 8.8% of variance in autism traits, 10% in repetitive behaviours, and 11.1% in social adaptive functioning. Similar variance was explained by FET subgrouping alone (SRS: 16.2%; ADOS: 17.6%; RBS: 9.2%; VABS: 16.7).

In previous studies, VIQ was found to be the strongest predictor of clinical features. [33]. Therefore, we also subdivided the autism group into IQ subgroups (above/below IQ 75) as a bar for comparison. The cut-off of 75 was used to correspond with the definition of our ID group in LEAP [26]. Subgrouping by IQ explained 10.2% of variance in current social symptoms (*t*(220) =  − 5.6, *p* < 0.001), 2.1% in autism traits (*t*(263) =  − 2.4, *p* < 0.05), 2.9% in repetitive behaviours (*t*(259) =  − 0.28, *p* < 0.05), and 11.5% in social adaptive functioning (*t*(247) = 5.7, *p* < 0.001). This shows that expression recognition subgroups explained more variance in clinical features than could be explained by IQ alone.

### Relationship between clusters and individual task performance, respectively, and neurofunctional differences

To externally validate the performance scores and clusters in terms of underpinning mechanisms, we investigated associations with neurofunctional responses during an established emotional face-matching task. The characteristics of participants with fMRI data are given in Table 4. Case–control comparisons revealed no significant between-group differences in amygdala or fusiform gyrus activation overall or within age groups (all *F* > 0.36 < 2.7, all *p* > 0.08).

**Table 4:** Participant characteristics for clustering participants with fMRI data

	Autism		Control	
	Cluster 1 *N* = 46	Cluster 2 *N* = 11	Cluster 3 *N* = 49	Cluster 4 *N* = 15
Age (yrs) Mean ± SD (Range)	18.14 ± 5.54 8.05–29.57	15.10 ± 6.15 7.41–27.88	17.2 ± 6.65 7.55–29.84	13.79 ± 4.48 9.27–27.11
IQ Mean (SD) Range	109.98 ± 13.88 85–148	92.91 ± 12.45 78–117	113.59 ± 10.69 96–142	102.67 ± 22.42 52–134
Sex m:f	28:18	10:1	30:19	12:3
ID participants	0 (0%)	0 (0%)	0 (0%)	3 (20%)
Neuropsychiatric symptoms*	15	4	0	1
Medication use	23	5	8	3

*Neuropsychiatric symptoms that data was gathered on included ADHD, anxiety, depression, and behavioural disorders

**Fig. 6:**
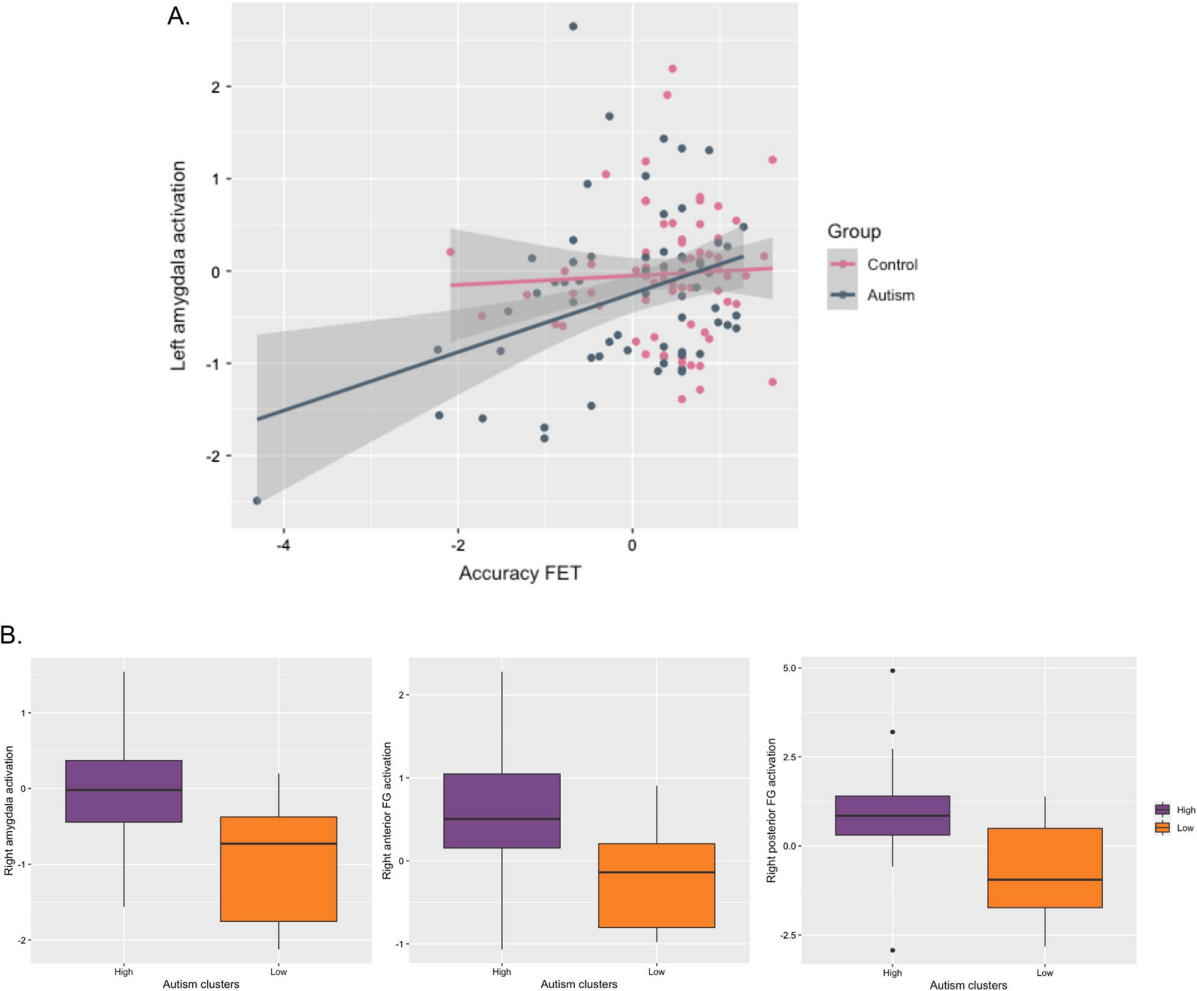
Relationship between expression recognition performance and functional activation. **A** FET performance and left amygdala activation **B** Clusters and right amygdala activation (left), clusters and right posterior FG activation (centre), clusters and right anterior FG activation (right)

In the autism group, accuracy on the FET was dimensionally related to increasing left amygdala activation (*r* = 0.24, *p* = 0.05) (Fig. 6A). Comparisons between clusters also showed that the autistic low-performing subgroup had on average significantly lower activation in the right amygdala (*F*(2, 64) = 5.3, *p*_corr_ < 0.05), left and right middle FG (left: *F*(2,65) = 5.9, *p*_corr_ < 0.05, right: *F*(2,65) = 9.16, *p*_corr_ < 0.01) and left and right posterior FG (left: *F*(2,66) = 6.3, *p*_corr_ < 0.01, right: *F*(2,66) = 5.3, *p*_corr_ < 0.05), than the autistic high-performing cluster. Reduced activation in the right amygdala (Fig. 6B) and right middle FG (Fig. 6C, D) explained 22.2% and 11.31% of variance, respectively. In the control group, we saw no significant dimensional relationships to functional activation or subgroup differences (all *F* < 0.042, *p* > 0.05).

## Discussion

This study investigated the role of expression recognition as a potential stratification biomarker for social communication difficulties in autism. We tested a large group of autistic individuals, diverse in age and intellectual ability, on three tests assessing expression recognition of simple and complex emotions with varying presentation times. In a first step, we examined group-level differences. We then explored which measure(s), alone or in combination using cluster analyses, were most sensitive to differences in clinical features and/or neurofunctional responses.

In line with previous studies, we found significant mean-group differences in accuracy across all three tests [13, 23, 24], with medium effect sizes similar to those reported in a previous meta-analysis [8]. In the autism group, difficulties were on average stronger on tests with complex emotions and/or brief presentation times, as indicated by somewhat higher effect sizes and, in some age groups, slower reaction times. However, contrary to the assumption that neurofunctional measures may be more sensitive than behavioural responses, in the present study we found only group-level differences in behavioural responses, but not in fMRI BOLD activity in regions previously associated with the recognition of facial emotion expressions.

Cluster analysis revealed two cluster solutions for both the autism and control groups. High agreement between hierarchical clustering and *K*-means (91.6%) indicated valid capture of the underlying data cluster structure. While both the majority of autistic (69.7%) and control participants (73.3%) fell in the high-performing clusters (the majority performing within or above 1 SD of the age-adjusted typically developing medians on at least two tests), 30.3% of autistic and 26.6% of control individuals fell in the low-performing clusters. Inspection of participant characteristics showed that the low-performing autistic cluster comprised individuals across all IQ levels (i.e. 27.7% had intellectual disability). Neuropsychiatric symptoms did not substantially affect expression recognition as 47.2% of autistic people in the low-performing cluster but also 34.9% of autistic people in the high-performing cluster had neuropsychiatric symptoms.

It should be noted that in the present study, we carried out clustering separately for the autism and control groups. This was motivated by our main goal to parse heterogeneity in expression recognition within the autism group, and to explore the clinical and functional relevance of expression recognition performance in autism. We repeated the approach in the control group to see whether there may be similar or different patterns of expression recognition performance across the three different tasks in non-autistic individuals. Although we found a two-cluster solution in both groups, the boundary threshold for ‘low-performing’ was somewhat more stringent in the autism group than the comparison group. Future studies may use the alternative, transdiagnostic approach of performing clustering on the combined sample and then characterise clusters in terms of potential enrichment by diagnostic groups.

Taken together, by comparing findings from our mean-group comparisons to the results from our cluster analyses, we highlight that mean-group differences are unsuited to making inferences about specific autistic individuals [22]. Although the autism group were on average less accurate in expression recognition than the control group, in fact only a proportion of autistic people—as well as a proportion of non-autistic people—had difficulties in expression recognition.

### Relationships to clinical features and neurofunctional underpinnings

A critical criterion for a test or measure to qualify it as a stratification biomarker consists of demonstration that it relates to—or predicts—a clinically relevant outcome. In the autism group, we observed significant subgroup differences in clinical features, which accounted for 9–14% of variance in autism traits, current severity of social difficulties and social adaptive behaviour. Previous studies suggested level of intellectual ability to be among the strongest predictors for functional outcomes in autism [33]. We therefore also subgrouped our autistic participants by IQ. This showed that subgrouping by behavioural expression recognition patterns explained 1.5–3 times more variance than was explained by IQ subgroups.

Furthermore, by relating behavioural expression recognition against neurofunctional activation during a fearful face-matching task, we showed that the autistic expression recognition subgroups significantly differed from one another in terms of functional activation patterns. More specifically, the autistic low-performing subgroup had significantly lower activation in the amygdala and fusiform gyrus bilaterally than the high-performing subgroup, explaining 20% and 10% of variance, respectively. This reaffirms the integral role of these structures in emotion recognition and provides a neurobiological substrate for the uncovered heterogeneity of emotion recognition in autism.

### Implications of findings for stratified or precision medicine approaches

Our finding that only subgroups of autistic and non-autistic people had behavioural difficulties in expression recognition, which were related to both clinical features and functional outcomes, highlights the need for more targeted ‘precision’ medicine or intervention approaches, rather than inferring treatment needs from the broad autism diagnosis alone.

First, we showed that not every autistic person requires support in expression recognition. In fact, the majority of autistic individuals appear to have no more difficulties in expression recognition than the majority of non-autistic individuals.

Second, analyses of neurofunctional responses indicate that the behavioural expression recognition difficulties we observed may be due to partly different mechanisms in autistic and non-autistic people. In the autism group, expression recognition difficulties were significantly related to lower amygdala and fusiform gyrus activation, while in the non-autistic group they were not. These findings should be treated as preliminary as they were based only on a subset of our participants who had completed all behavioural tests and the fMRI paradigm, and they were largely restricted to people without ID. However, if replicated, they may indicate that autistic and non-autistic subpopulations may have expression recognition difficulties for different reasons, and therefore may benefit from different behavioural or pharmacological interventions to increase their ability to recognise emotions from faces.

For measures of expression recognition to be used as (multi- or univariate) stratification markers in future clinical settings, we sought to empirically derive cut-off values. Emphasis on sensitivity vs. specificity (and thus stringency of cut-off values) depends on investigators’ or clinicians particular context of use, such as the cost of including individuals in a stratified clinical trial and their likely benefits [34]. Moreover, from a practical perspective (e.g. costs, participant burden) it is useful to know whether multi-variate markers composed of performance on different measures have significantly greater accuracy than univariate markers based on a single index. We showed that of the three tests used, FET performance had highest accuracy in assigning individuals to the high vs. low-performing subgroups. This suggests that in the current instance FET performance alone (univariate marker) approximated the information provided by multi-variate assessments (clustering from 3 tests).

### Limitations

First, our findings will require independent replication—not only of cluster assignments/composition, but critically also of the relationship between clusters and clinical and neurofunctional features. Independent replication is often considered as a gold standard. It was not possible here as we are not aware of any current dataset that includes the relevant measures. However, we demonstrated internal robustness of results by only including participants who were consistently assigned to the same subgroup using two clustering approaches with fundamentally different assumptions. We argue that internal robustness—that is, generating identical results with different methods on the same data—is a critical though frequently understudied requirement before moving on to external replication (different methods, different data and likely participant characteristics).

A second limitation was that we had different sample sizes for different analyses. While we used the total number of participants who had completed a given task for the group comparisons of each to maximise power, numbers were reduced for clustering analyses by only including participants who had completed all three behavioural tasks. However, we replicated the mean-group findings in this smaller group. Furthermore, in the external validation study, only a subset of participants participated in the fMRI experiment, and per protocol this excluded participants with mild ID, to reduce participant burden as they had also completed another MRI session. Although the sample size (*N* = 137) was high for a task-related fMRI study in autism research, validation of the brain-behaviour associations was limited by the sizes of the low-performing clusters.

Third, future work should further optimise and standardise behavioural facial expression recognition tasks. The three tests included in this study were chosen because they tested different aspects of expression recognition, showed at least adequate performance characteristics (such as test–retest reliability), and/or obtained large effect sizes [13]. However, further indicators relevant for clinical use, such as sensitivity to change, remain to be established. We found the FET to be most sensitive in subgroup assignments, and to account for the highest variance in clinical symptoms and functional activation. As effect sizes were comparable to those attained in the combined clusters, from a practical perspective, the FET alone may be carried forward as a proxy marker. The FET was less sensitive in children than adolescents/adults, possibly because a proportion of TD children also showed difficulties. Future task optimisation should aim to increase discriminant validity, particularly in children. Standardisation and creation of normative ranges will also help to interpret individual test scores based on age/ability level, or other demographic variables.

## Conclusions

In a large and heterogeneous autism sample, we found that around 30% of autistic individuals had difficulties with expression recognition, which were related to both clinical features and neurofunctional differences. This indicates that a subset of autistic individuals may benefit from targeted support or interventions in expression recognition. Standardisation of tasks and replication of findings will be necessary to further validate expression recognition as a clinically relevant stratification biomarker for autism.

## Supplementary Information

**Additional file 1.** Supplementary materials.

## Data Availability

The datasets generated and/or analysed during the current study are not publicly available due to an embargo period but are available from the corresponding author on reasonable request.

## Abbreviations

KDEF: Karolinska Directed Emotional Faces
RMET: Reading the Mind in the Eyes Task
FET: Films Expression Task
LEAP: Longitudinal European Autism Project
TD: Traditionally developed
ID: Intellectual disability
ADI-R: Autism Diagnostic Interview-Revised
ADOS-2: Autism Diagnostic Observation Schedule 2
CSS-SA: ADOS Calibrated Severity Score for Social Affect
SRS-2: Social Responsiveness Scale Second Edition
RBS-R: Repetitive Behavior Scale-Revised
VABS: Vineland Adaptive Behavior Scales-2nd Edition
DAWBA: Development and Wellbeing Assessment probability bands
ART: Accuracy-adjusted response times
ROI: Regions of interest
FG: Fusiform gyrus

## Footnotes

1 We use the term autism here in favour of the official term autism spectrum disorder to reflect the preference by several members of the autism community that advise on AIMS-2-TRIALS to stress differences over deficits.
