# Supplementary material for: Facial expression recognition is linked to clinical and neurofunctional differences in autism
Source: Mol Autism. 2022 Nov 10;13:43. doi: 10.1186/s13229-022-00520-7 (PMC9650909; doi:10.1186/s13229-022-00520-7)
Supplement: Supplementary file 1 — Additional file 1. Supplementary materials. [file 13229_2022_520_MOESM1_ESM.docx]

**Supplementary Materials 1: Task description, example stimuli and procedures**

The *Karolinska Directed Emotional Faces task (KDEF)*

The KDEF tests for recognition of basic emotions and allows for long presentation times. We used an adapted version with 70 trials (3), reduced from the original 140 trials, to reduce assessment time. In each trial, participants are shown a photograph of a person’s face and asked to select which of seven words (happy, sad, angry, surprised, afraid, disgusted, or neutral) best describes the expression displayed. The maximum time allotted to each participant per trial is twenty seconds. Should they not respond within this time frame, the trial counts as failed. The stimuli used in the KDEF have previously been validated on emotional content, intensity and arousal, and good test–retest reliability of .88 has been reported (4).


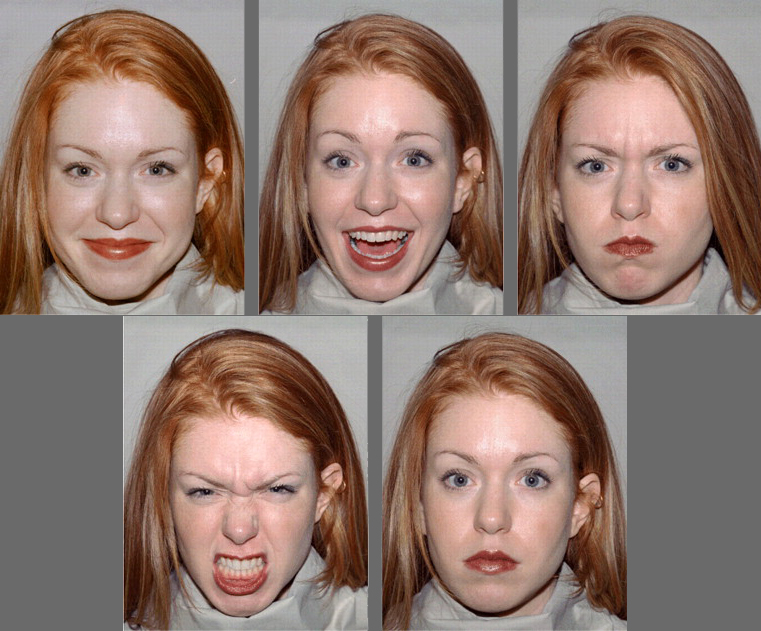


The *Reading the Mind in the Eyes test (RMET)*

In the RMET (5) participants are asked to identify complex emotions and mental states. The participant is presented with an image of only the eye portion of a face (male or female), together with four descriptors. All four of these were selected to have similar emotional valence. Before performing the task, participants were provided with a list of all the terms along with their dictionary definitions. They were encouraged to read the definition of a term should they be unfamiliar with it and could consult this list again at any time. Participants received either an adult (36 items), adolescent (31 items) or child (28 items) version of the test depending on age (adults 18-30, adolescents 12-17, children 6-11) and ability level. Most adolescents and adults with ID (with/without autism) completed the child version, in order to be consistent with their mental age. All participants who performed an age-inappropriate version of the test were excluded. Test-retest reliability of .7 was found in a French version of the task (6).


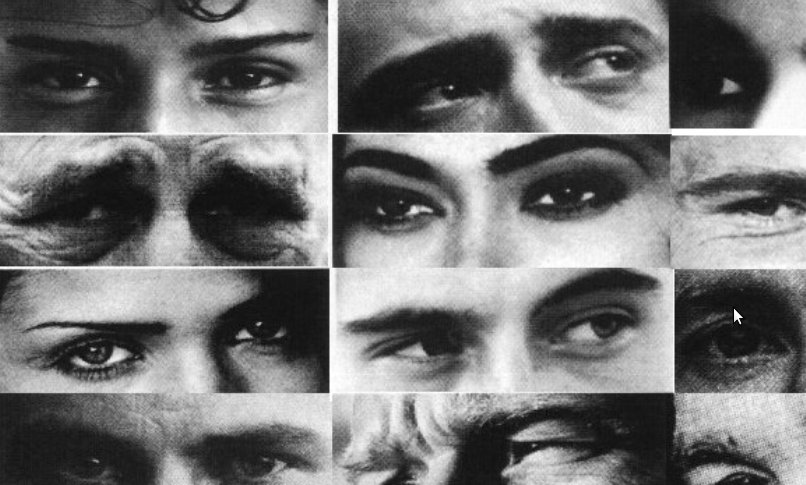


*The Films Expression Test (FET)*

This task (7) aims to reflect characteristics of real-life expression recognition through briefly presented still images from movie scenes. Participants are shown a target emotion, then three images of the same actor displaying different expressions one after the other. They are subsequently asked to indicate which image best describes the target emotion. The task includes a large range of both basic and complex emotions. It differs from both the KDEF and the RMET in this mixture of both emotion types, as well as in its shorter presentation times (500ms). Taken together, this task aims to emulate a real-life social interaction, where changes in emotion happen quickly.

Participants were given either an adult version of the task (56 items, 2 practice items) or a child version (36 items, 2 practice items). Adolescents and adults were presented with a vocabulary list to ensure comprehension of all terms used. Before completing the FET, children played a *vocabulary identification game* in which they were asked to fill in the correct emotional descriptor for an imaginary situation to ensure that they understood the target words. We excluded trials with vocabulary items that participants did not understand.

For the most part, participants with mild ID were given the child version of the task, as it was judged that their mental age would be closer to that of the child group. There were 20 children who performed the adult version of the task, and three participants with ID who performed the adult version of the task but performed the vocabulary identification game the children were given. For these 23 participants, we analyzed only the items common between the child and the adult version of the task and integrated them into the analysis of the child version.

Participants also completed a Rapid Visual Processing control task in which they had to identify which of briefly (500ms) presented shapes or fruit best matched a target word (e.g., apple) to control for the possibility that difficulties on the main task are due to broader difficulties with rapid information processing. The adult version of the task has previously been shown to have acceptable test-retest reliability (ICC=. 74, (7)).


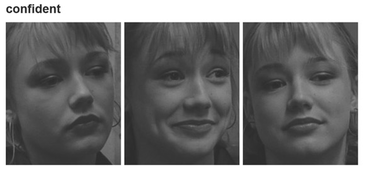


Across all three tasks, participants were always instructed to select the appropriate descriptor for an emotional face as quickly and accurately as possible. 1.96% of individuals in the KDEF, 4.31% in the RMET and 6.27% in the FET were excluded upon the Quality Assurance/Quality Control procedures described above.

**Supplementary Materials 2: Details of clustering analysis**

Cluster analysis was carried out using both hierarchical clustering (Ward’s linkage, Euclidean distance) and Kmeans (25 random starts). These two methods were chosen because they represent the most common variants of centroid and connectivity-based clustering, respectively, which are based on different assumptions and tend to produce the most divergent results.

The methods were both run using 2 to 9 cluster numbers and the optimal number of clusters was defined as the maximal intersection between the clusters found by the two methods. Cluster stability was estimated by drawing 1000 bootstrap samples from the full data set, running the two clustering methods on each sample and determining the number of times that two subjects were in the same clusters with both methods. The resulting n x n stability matrix (n is the number of subjects) was then thresholded by subtracting the frequency of co-clustering obtained using randomly permuted output from the two clustering methods. The software was written as an R script executed in Rstudio.

To calculate thresholds for multivariate clusters, we performed hundred Gaussian shells around the typically developing group (excluding those with ID) using the Hypervolume package in R. Hypervolume constructs a hypervolume by building a Gaussian kernel density estimate on an adaptive grid of random points wrapping around the original data points. The bandwidth vector reflects the axis-aligned standard deviations of a “hyperelliptical kernel”. The resulting hypervolume is thresholded to include 50% (light grey in Figure 1), 95%, 99% and 99.9% of the 50,000 random points in the hypercloud, and the resulting thresholded volumes are used to compute convex hulls, which are then transformed to surface meshes and represented as decreasingly transparent grey shells. The data from the Autism and control group (TD +ID) were then separately inserted into the graphics using colours indicating cluster membership.

**Figure 1** shows a 3D representation of a Gaussian hypervolume with participants in the high (green) and low (red) performing clusters for the Autism (**A**) and control groups (**B**). At 95% probability, 98.8% of the autistic high-performance cluster and 26.3 % of the low performance cluster were included.

X-axis age, Y-axis KDEF, and Z-axis FET (RMET not included). The green dots represent participants in the “high expression recognition performance cluster” The red dots indicate that the majority of participants in the “low expression recognition performance” cluster performed outside of the normative shell at p <0.001. Black dots indicate inconsistently clustered participants.

A. Autism group


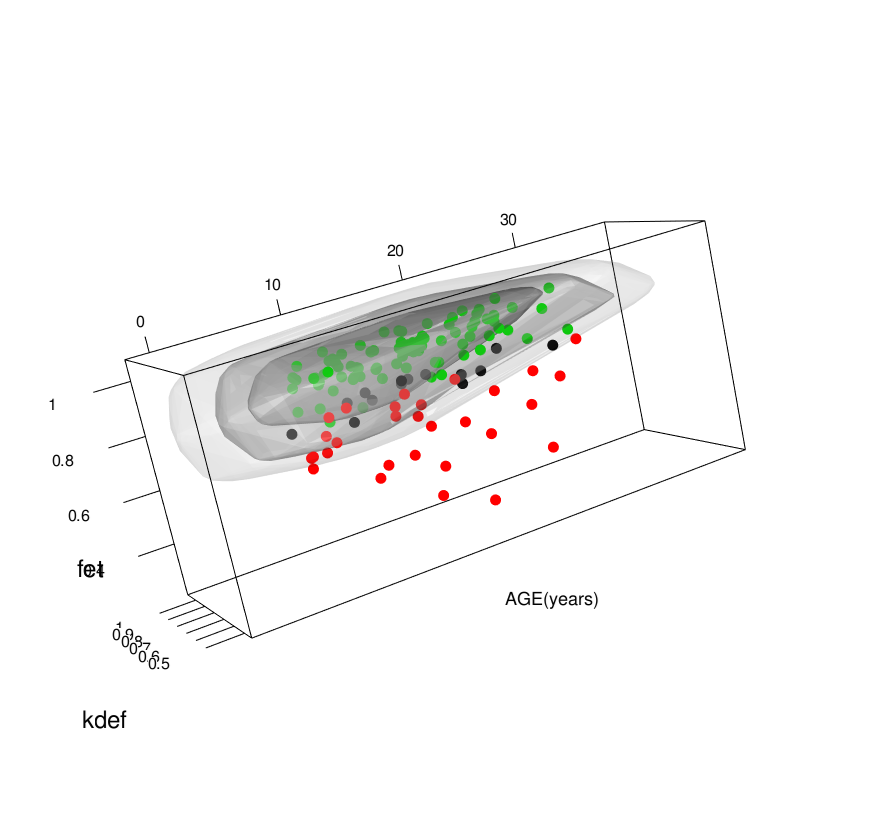


B. Control group


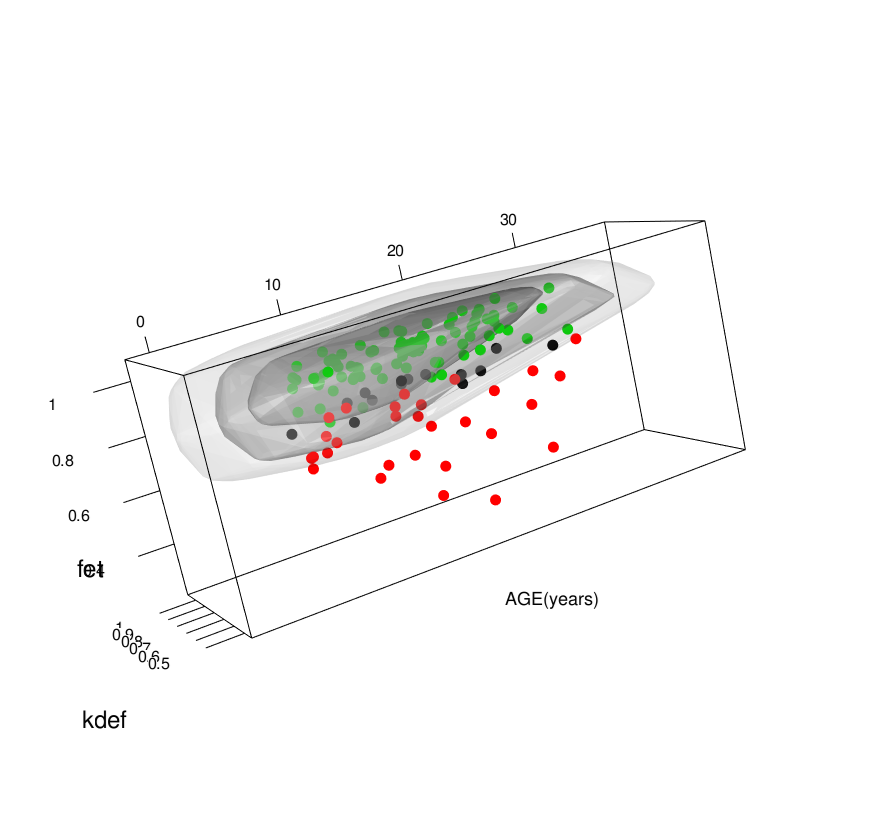


To estimate sensitivity and specificity of classifying individuals into high- vs. low-performing subgroups from single task performance at different cut-offs (1, 1.5, 2 and 3Ds) we generated RoC curves by treating the low-performance cluster as false positives in the Gaussian envelope and the high-performance cluster as true positives.

**Figure 2** shows RoC curves generated to estimate sensitivity and specificity of classifying individuals into high- vs. low-performing subgroups from single task performance at different cut-offs (1, 1.5, 2 and 3Ds). At 1 SD FET performance alone had 95% sensitivity and 100% specificity and at 2 SD 98% sensitivity and 87% specificity in assigning autistic individuals to high vs. low-performing subgroups, KDEF approximately 95 % sensitivity and 92% specificity at 1SD and 95% and 76% at 2SD. In comparison, RMET only had approximately 84% sensitivity and 75% specificity at 1SD and 94% sensitivity and 37% specificity at 2 SDs. Hence, FET performance alone had high sensitivity/ specificity in assigning individuals to the low-performing cluster.


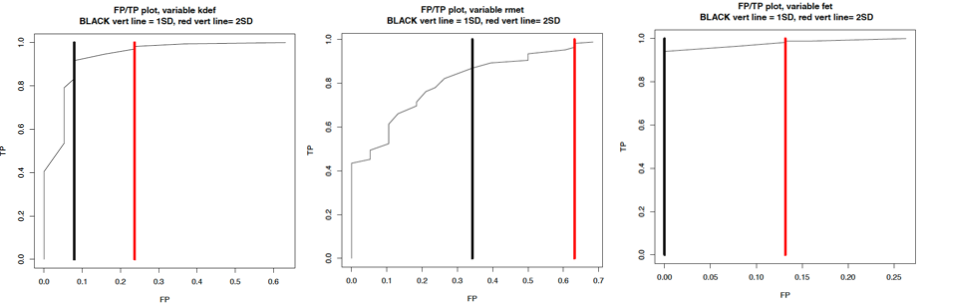


**S**ensitivity and specificity in assigning individuals to high vs. low-performing subgroups at different cut-offs (vertical lines denote 1 and 2 SDs below the TD medians), in the order: KDEF, RMET, FET

**Supplementary Materials 3: fMRI paradigm, acquisition parameters, pre-processing and analyses**

Neuroimaging data was collected at T1 (T2 data were collected but unavailable at the time of writing) during an emotional face matching task, which has been shown to reliably elicit brain responses in ventral visual areas extending into the amygdala in neurotypical groups ((8), (9)). Participants first had the opportunity to practice the emotional face matching task in a ‘mock scanner’ session, to ensure that they fully understood task instructions and procedures and were familiarised with the scanning environment. 

In the experimental condition, participants were shown three black-and-white emotional faces depicting fear or anger. Participants were asked to match the face shown in the top row to one of two faces in the bottom row (identity match), using the left or right button on an fMRI compatible response device. In the control condition, faces were replaced by black-and-white oval shapes. Faces and shapes trials (5s) were presented in alternating blocks, each consisting of six trials, with four blocks presented for each condition (4min 28s). The emotional face matching task was administered at the end of a 60-minute scanning protocol. Stimuli were streamed via Presentation® software and the researcher delivered instructions to the participant during the scan, as follows: 

*”In the next task, you have to match shapes and faces. If the upper picture matches the lower left picture, you have to press the left button with your first [i.e. index] finger. If the upper picture matches the lower right picture, you have to press the right button with your middle finger.”*

Across both conditions, behavioural accuracy and reaction time (RT) were recorded

To limit the overall scan time for children and those with mild ID, as per protocol, the majority of participants (75.9%) were adolescents and adults, and only individuals with IQ in the normal range (Schedules A-C).

Data were acquired on 3 Tesla MRI scanners and acquisition protocols were harmonized across sites as closely as possible. BOLD fMRI was collected using an echo-planar-imaging sequence with the following parameters: TR 2000 ms, TE 30 ms, 28 oblique slices per volume, 4 mm slice thickness, 1 mm slice distance, 80° flip angle, 192 mm FOV, and 64 × 64 matrix. Data were subjected to an extensive quality assessment pipeline (1).

*fMRI data analysis*

Functional imaging data were pre-processed using standard analysis routines implemented in SPM12 (http://www.fil.ion.ucl.ac.uk/spm/), including slice-time correction, a two-step realignment procedure, unified segmentation and normalization to standard stereotactic space as defined by the Montreal Neurological Institute (MNI), and smoothing with an 8mm full-width-at-half-maximum Gaussian Kernel.

A general linear model (GLM) was defined for each subject that included the boxcar reference vectors for the two task conditions (convolved with the standard SPM hemodynamic response function) and the 6 head motion parameters from the realignment step as covariates of non-interest to account for residual motion. During model estimation, the data were high-pass filtered (cut-off: 128 seconds). To identify brain regions that show a stronger response to emotional face stimuli compared to geometric shapes, individual maps of the contrast “matching faces > matching shapes” were computed. Activation parameters were extracted from a-priori defined regions of interest (ROI) in the amygdala and fusiform gyrus. ROI masks were defined using Anatomical Probability Maps (Anatomy toolbox) for the amygdala, posterior (area FG2) and middle (area FG4) fusiform gyrus for each hemisphere. A manuscript reporting on condition and group analyses in the entire LEAP sample is currently in preparation (Moessnang et al., in preparation).


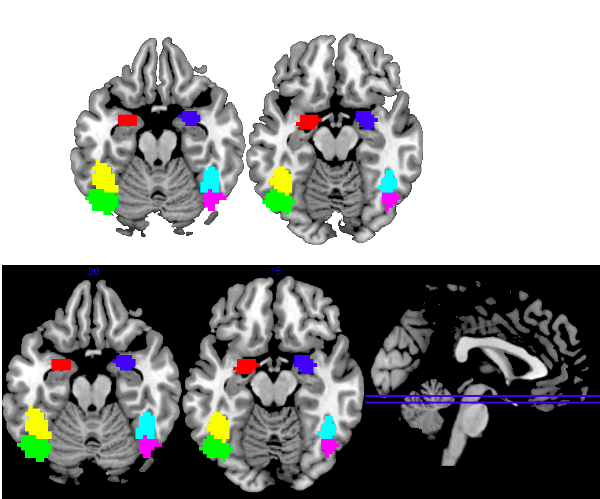

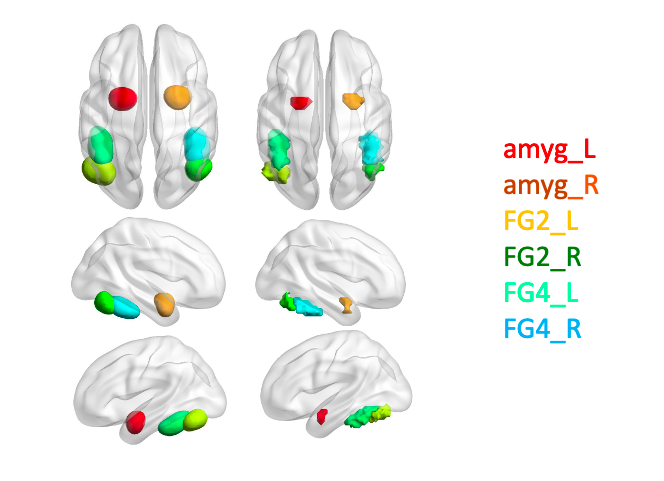


We excluded participants with excessive head motion defined as mean framewise displacement > 0.5 mm (Jenkinson et al., 2002) and region-specific signal loss > 0.5. The latter led to the exclusion of 4 autistic individuals in the left amygdala, 2 in the right amygdala and 1 in the middle fusiform gyrus (FG), and in the comparison group 2 in the left amygdala ROI, 1 in the right, 1 in the left posterior FG and 3 in the right middle FG. The total sample comprised between 128 and 134 participants, depending on the region, who did not significantly differ in terms of head motion (t(1,133)=.4, p=.52). We used ANCOVAs or logistic regression to compare the autism and control group, as well as the clusters/ subgroups, on regional functional activation. We found no case-control group-level differences in any ROI (all p >.05). Partial correlations were used to test for dimensional relationships with FET performance. In all analyses, study site (to account for scanner differences) and head motion were used as covariates. Bonferroni corrections were used to adjust significant thresholds by the number of ROIs tested (0.05/6=0.0083).

1. Loth E, Charman T, Mason L, Tillmann J, Jones EJH, Wooldridge C, et al. (2017): The EU-AIMS Longitudinal European Autism Project (LEAP): design and methodologies to identify and validate stratification biomarkers for autism spectrum disorders. *Mol Autism*. 8:24.

2. Charman T, Loth E, Tillmann J, Crawley D, Wooldridge C, Goyard D, et al. (2017): The EU-AIMS Longitudinal European Autism Project (LEAP): clinical characterisation. *Mol Autism*. 8:27.

3. Sucksmith E, Allison C, Baron-Cohen S, Chakrabarti B, Hoekstra RA (2013): Empathy and emotion recognition in people with autism, first-degree relatives, and controls. *Neuropsychologia*. 51:98-105.

4. Goeleven E, De Raedt R, Leyman L, Verschuere B (2008): The Karolinska directed emotional faces: a validation study. *Cognition and emotion*. 22:1094-1118.

5. Baron-Cohen S, Wheelwright S, Hill J, Raste Y, Plumb I (2001): The "Reading the Mind in the Eyes" Test revised version: a study with normal adults, and adults with Asperger syndrome or high-functioning autism. *J Child Psychol Psychiatry*. 42:241-251.

6. Prevost M, Carrier M-E, Chowne G, Zelkowitz P, Joseph L, Gold I (2014): The Reading the Mind in the Eyes test: validation of a French version and exploration of cultural variations in a multi-ethnic city. *Cognitive neuropsychiatry*. 19:189-204.

7. Loth E, Garrido L, Ahmad J, Watson E, Duff A, Duchaine B (2018): Facial expression recognition as a candidate marker for autism spectrum disorder: how frequent and severe are deficits? *Mol Autism*. 9:7.

8. Hariri AR, Tessitore A, Mattay VS, Fera F, Weinberger DR (2002): The amygdala response to emotional stimuli: a comparison of faces and scenes. *Neuroimage*. 17:317-323.

9. Plichta MM, Schwarz AJ, Grimm O, Morgen K, Mier D, Haddad L, et al. (2012): Test-retest reliability of evoked BOLD signals from a cognitive-emotive fMRI test battery. *Neuroimage*. 60:1746-1758.

**Supplementary Materials 4.** Correlations between performance (accuracy, ART) on each expression recognition task, by group (Left panel: Autism; right panel: Control group)

| **Autism**  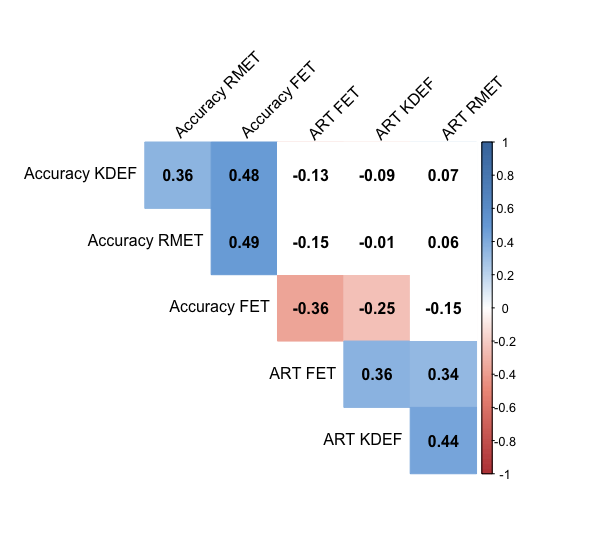 | **Control group**  **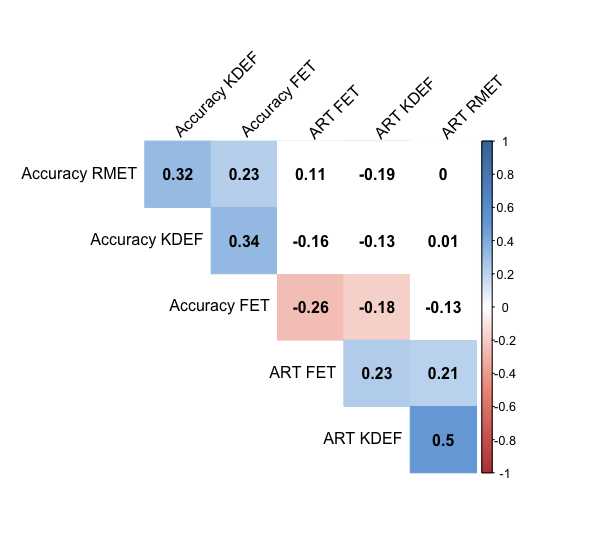** |
| --- | --- |
